# Developing a primary tumor and lymph node 18F-FDG PET/CT-clinical (TLPC) model to predict lymph node metastasis of resectable T2-4 NSCLC

**DOI:** 10.1007/s00432-022-04545-6

**Published:** 2022-12-24

**Authors:** Meng Wang, Liu Liu, Qian Dai, Mingming Jin, Gang Huang

**Affiliations:** 1 School of Health Science and Engineering, University of Shanghai for Science and Technology, Shanghai, 200093 China; 2 Shanghai Key Laboratory of Molecular Imaging, Jiading District Central Hospital Affiliated Shanghai University of Medicine and Health Sciences, Shanghai, 201318 China; 3 Department of Nuclear Medicine, Shanghai Chest Hospital, Shanghai Jiao Tong University, Shanghai, 200003 China; 4 Shanghai Key Laboratory of Molecular Imaging, Zhoupu Hospital, Shanghai University of Medicine and Health Sciences, Shanghai, 201318 China

**Keywords:** Radiomics-clinical, Machine learning, NSCLC, Lymph node metastasis, ^18^F-FDG PET/CT

## Abstract

**Purpose:**

The goal of this study was to investigate whether the combined PET/CT radiomic features of the primary tumor and lymph node could predict lymph node metastasis (LNM) of resectable non-small cell lung cancer (NSCLC) in stage T2-4.

**Methods:**

This retrospective study included 192 NSCLC patients who underwent tumor and node dissection between August 2016 and December 2017 and underwent ^18^F-fluorodeoxyglucose (^18^F-FDG) PET/CT scanning 1–3 weeks before surgery. In total, 192 primary tumors (> 3 cm) and 462 lymph nodes (LN > 0.5 cm) were analyzed. The pretreatment clinical features of these patients were recorded, and the radiomic features of their primary tumor and lymph node were extracted from PET/CT imaging. The Spearman’s relevance combined with the least absolute shrinkage and selection operator was used for radiomic feature selection. Five independent machine learning models (multi-layer perceptron, extreme Gradient Boosting, light gradient boosting machine, gradient boosting decision tree, and support vector machine) were tested as classifiers for model development. We developed the following three models to predict LNM: tumor PET/CT-clinical (TPC), lymph PET/CT-clinical (LPC), and tumor and lymph PET/CT-clinical (TLPC). The performance of the models and the clinical node (cN) staging was evaluated using the ROC curve and confusion matrix analysis.

**Results:**

The ROC analysis showed that among the three models, the TLPC model had better predictive clinical utility and efficiency in predicting LNM of NSCLC (AUC = 0.93, accuracy = 85%; sensitivity = 0.93; specificity = 0.75) than both the TPC model (AUC = 0.54, accuracy = 50%; specificity = 0.38; sensitivity = 0.59) and the LPC model (AUC = 0.82, accuracy = 70%; specificity = 0.41; sensitivity = 0.92). The TLPC model also exhibited great potential in predicting the N2 stage in NSCLC (AUC = 0.94, accuracy = 79%; specificity = 0.64; sensitivity = 0.91).

**Conclusion:**

The combination of CT and PET radiomic features of the primary tumor and lymph node showed great potential for predicting LNM of resectable T2-4 NSCLC. The TLPC model can non-invasively predict lymph node metastasis in NSCLC, which may be helpful for clinicians to develop more rational therapeutic strategies.

## Introduction

Lung cancer is the leading cause of cancer-related deaths worldwide, accounting for almost one-fifth of all cancer-related deaths (Sands et al. 2021). Non-small cell lung cancer (NSCLC) accounts for over 80% of lung cancer subtypes and has a 5-year survival rate of about 10–15% for all stages (Siegel et al. 2021; Goldstraw et al. 2011). Although more than half of the NSCLCs are first diagnosed with local or distant metastasis and therefore are past the point of surgery, tumor resection is still the first option for lung cancer treatment (Maniwa et al. 2020) of those with a resectable primary tumor and without lymph node metastasis (N0), those with only with local lymph node metastasis (limited in ipsilateral pulmonary and ipsilateral mediastinum or submarine, N1-2), and those without distant organ metastasis (M0). For patients with distant lymph node metastasis (N3), nonsurgical treatment (e.g., chemo-/radio-therapy) instead of tumor resection is recommended. Therefore, accurate lymph node (N) staging is essential for developing different treatment strategies for resectable NSCLC (Ettinger et al. 2021).

Preoperative invasive lymph nodes biopsy and pathology is the gold standard for assessing LNM of lung cancer patients. However, this approach is not effective for many patients for the following reasons: (1) due to multiple suspected metastatic lymph nodes in the mediastinum and hilus pulmonic, the selection of lymph nodes is difficult during biopsy, and some positive lymph nodes may be omitted, which would result in a false-negative report; (2) for technical reasons, invasive examination samples may be too small, resulting in failure of pathological examination; and (3) the patient is mentally or physically unable to tolerate invasive biopsy. Promisingly, noninvasive ^18^F-FDG PET/CT has exhibited great potential for non-invasively predicting LNM in may cancer types, including NSCLC (Szlubowski et al. 2014; Roberts et al. 2000; Torigian et al. 2007; Park et al. 2020; Wang et al. 2017; Terán and Brock 2014). Therefore, this technique may help clinicians make more rational treatment decisions for patients without pathologic data about suspicious lymph nodes. However, the false-positive rate of ^18^F-FDG PET/CT is still high in detecting malignancy in normal-sized lymph nodes and in ruling out malignancy in patients with coexisting inflammatory or infectious diseases, which hampers its application to N staging of lung cancer (Roberts et al. 2000). Additionally, due to the limited number and diversity of image features, the thresholding strategy is mainly based on thresholding of maximum standardized uptake value (SUV_max_) or mean standardized uptake value (SUV_mean_). To date, the diagnostic power of ^18^F-FDG PET/CT has not been fully explored (Turkmen et al. 2007), and developing more reliable methods based on PET/CT to accurately predict LNM of NSCLC would be an important advance in diagnostic techniques.

High-throughput radiomics has recently emerged as a powerful approach for identification of imaging biomarkers that can be used to build decision-support systems for cancer treatment (Hyun et al. 2019^) (^Lee et al. 2015). Machine learning can be significantly effective for object detection and classification, and it is being increasingly used to help clinicians predict LNM based on radiomic features of primary tumors or lymph nodes (Goldstraw et al. 2016; Cong et al. 2020a; Ouyang et al. 2021; Zheng et al. 2021; Scrivener et al. 2016; Li et al. 2015) with area under the curve (AUC) ranging from 0.77 to 0.86_._ However, few studies have combined radiomic features of primary tumor and lymph node extracted from PET/CT images to build a LNM prediction model by applying radiomics and machine learning (ML). It remains unclear whether the radiomics-clinical features combined primary tumor with lymph nodes is an effective method to improve the PET/CT’s efficacy in predicting LNM of resectable T2-4 NSCLC.

The risk of LNM increases with the growth of primary tumor size: the incidence of LNM in resectable T2-4 NSCLC is about 50%, whereas it is only about 15% in T1 NSCLC (Xue et al. 2623; Chen et al. 2019a; Moulla et al. 2019). In this study, we presented a comprehensive analysis of the radiomic features of primary tumors and lymph nodes based on PET/CT images and the clinical features in 192 resectable T2-4 NSCLC. Our goal was to construct and validate a tumor and lymph PET/CT-clinical (TLPC) model capable of predicting LNM in T2-4 NSCLC, which is the relatively high-risk group for LNM. Ultimately, this new method may improve the efficiency of preoperative N staging for NSCLC.

## Patients and methods

### Patients

**Fig. 1:**
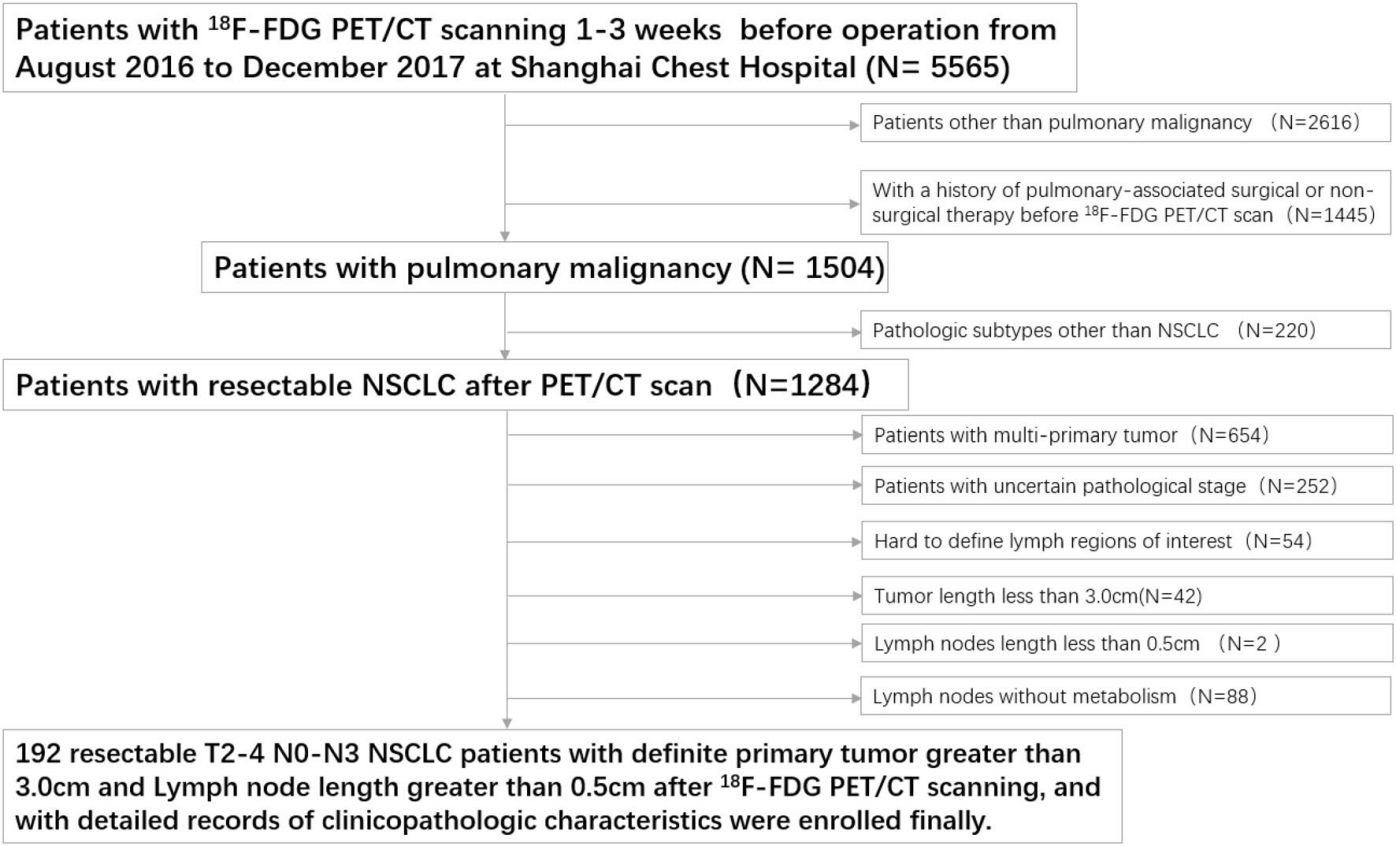
Patient selection flowchart

This study retrospectively reviewed the charts of 5565 patients examined by ^18^F-FDG PET/CT scanning 1–3 weeks before surgery between August 2016 and December 2017 at Shanghai Chest Hospital and identified 192 pulmonary malignancy patients with resectable T2-4 NSCLC. The exclusion criteria were as follows: (1) patients with other than pulmonary malignancy (2616 cases); (2) a history of pulmonary-associated surgical or non-surgical therapy before the ^18^F-FDG PET/CT scan (1445 cases); (3) pathologic subtypes other than NSCLC (220 cases); (4) multi-primary tumors (654 cases); (5) an uncertain pathological stage (252 cases); (6) hard to define lymph regions of interest (54 cases); (7) tumor length < 3.0 cm (42 cases); (8) lymph node length < 0.5 cm (2 cases); and (9) lymph nodes without metabolism (88 cases). All patients involved underwent lobectomy combined with systematic hilar (N1) and mediastinal lymph (N2) node dissection within 3 weeks after ^18^F-FDG PET/CT examination. The pathological mediastinal lymph node status involved in this study is post-operative pathological N (pN) staging according to the post-operative pathological results. According to the pN staging, the included cases were divided into negative (pN0-1) and positive (pN2) groups. The process of case screening and grouping is shown in Fig. 1. This retrospective study was reviewed and approved by the Institutional Review Board of Shanghai Chest Hospital and the requirement for informed patient consent was waived.

### PET/CT scanning

All patients selected in this study underwent the same PET/CT examinations by using the same equipment (Biograph mCT-S PET/CT (64-slice spiral CT), Siemens, Munich, Germany). ^18^F-FDG was produced and supplied by Shanghai Atom Kexin Pharmaceutical Co., Ltd. (Shanghai, China), with a pH value of ~ 7.0 and radiochemical purity of > 95%. Before the scan, the patients fasted for at least 6 h and maintained a blood glucose level < 7.8 mmol/L. According to each patient’s weight, the amount of ^18^F-FDG injected was based on each patient’s weight according to the standard of 0.10–0.15 mCi/kg. It was injected during a period of calm rest for 45–60 min. The parameters of the CT scan were set with the tube voltage of 120 kV, the tube current adjusted using CARE Dose technology. And the CT images were reconstructed to a 512 × 512 matrix corresponding to a 1 mm pixel size with thickness of 2 mm, 0.98 mm in-plane spatial resolution. The PET scan was performed after the CT scan finished. The collection of PET images was set in 5–6 beds at 2 min per bed position, and then the images were reconstructed to a 200 × 200 matrix corresponding to a 4 mm pixel size with thickness of 3 mm, 4.07 mm in-plane spatial resolution. No low-pass, smoothing filter was applied to the images after reconstruction. The PET images were attenuated by CT data and reconstructed by TrueX + TOF method.

### Pathological diagnosis

All post-operative pathological sections from patients selected in this study were reviewed by an experienced pathologist (Yichen Han, > 15 years of experience with lung cancer pathologic diagnosis). The pathological mediastinal lymph node status was also recorded.

### Image preprocessing

The target lesions in this study were primary tumors and the lymph nodes with ^18^F-FDG uptake. The volume of interest (VOIs) were semi-automatically segmented around the tumor outline to identify the largest cross-sectional area of the target lesions on the fusion PET/CT images. VOIs were further reviewed and corrected by two nuclear radiologists (Gang Huang and Liu Liu. > 15 years of experience) who were blinded to the pathologic or radiologic information. SUV_max_ > 30% was used as the SUV threshold to determine the final contouring margins of the target. Any different opinions were settled by consensus.

### Feature extraction

For an accurate diagnosis, more texture features and digital information needed to be extracted from PET/CT images. All data were standardized and normalized to facilitate the statistical analysis of index evaluation values. In total, 2662 features were extracted, including 2436 CT-features of primary tumor and lymph node extracted using the PyRadiomics platform. The features were developed to standardize the calculation of the radiomic feature algorithms and ease the feature extraction process to improve reproducibility of the findings (Griethuysen et al. 2017). Additionally, 216 PET-features of primary tumors and lymph nodes were automatically extracted using the Chang Gung Image Texture Analysis package in MATLAB 2012a (MathWorks Inc., Natick, MA, USA) (Fang et al. 2014). The CT-features were extracted based on the original image and by applying Laplacian of Gaussian and wavelet filters. To extract the PET-features, the SUV values contained within the ROIs were relatively resampled to 64 different values to yield a limited range of values; this was done to reduce the noise and to normalize the images (Yang et al. 2017).

### Feature selection

**Fig. 2:**
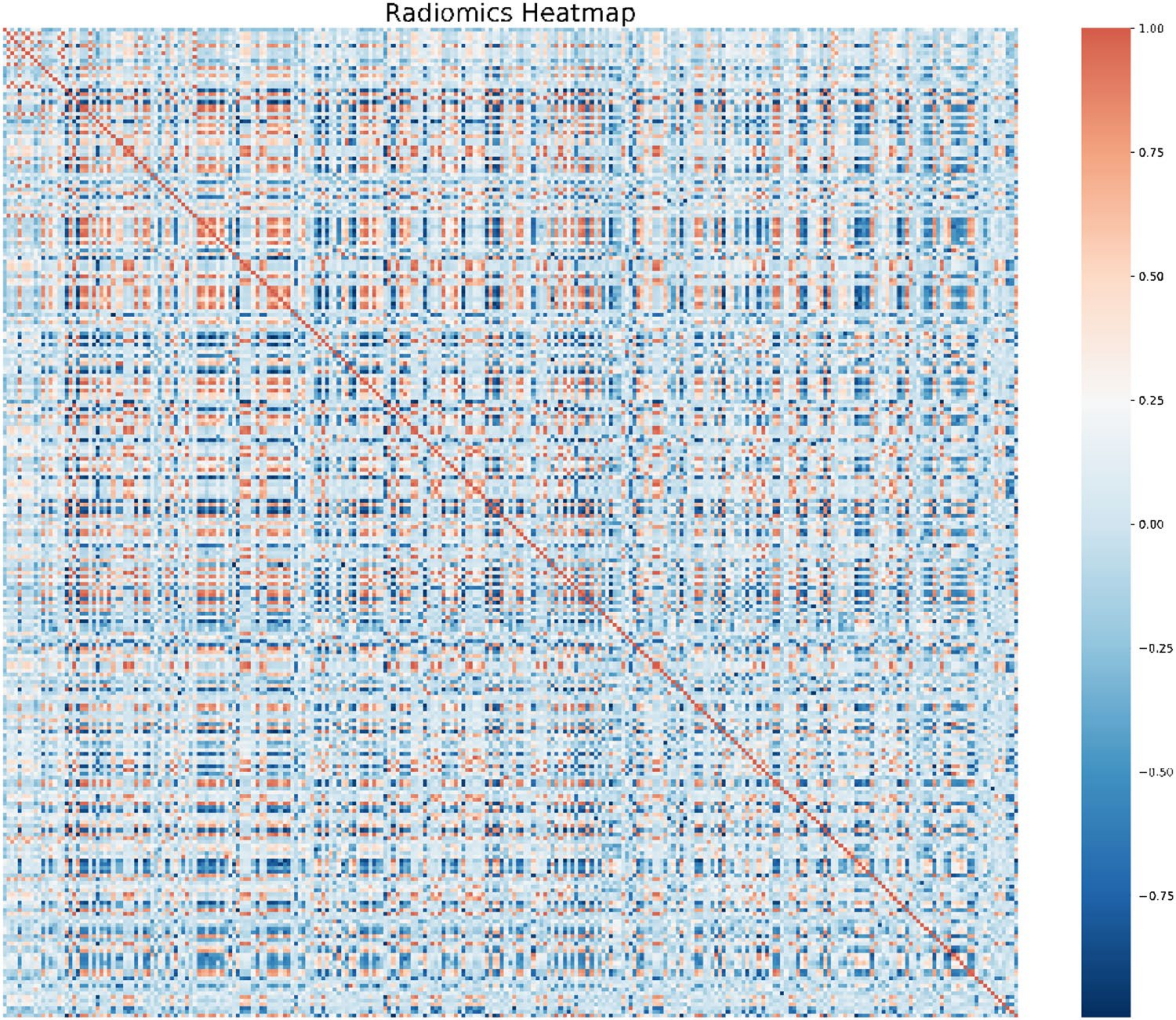
Radiomics heat map showing the Spearman correlation coefficient among the top 260 radiomic features

**Fig. 3:**
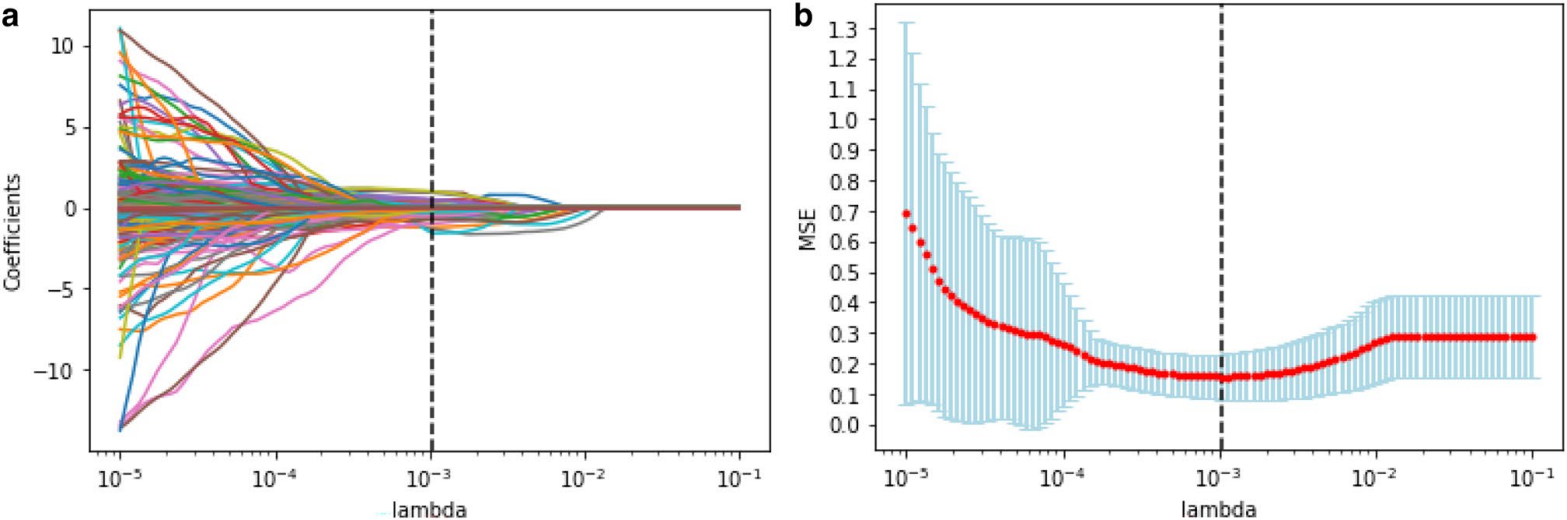
Radiomic feature selection via LASSO regression. **a** LASSO coefficient profiles of the 50 candidate radiomic features. **b** Model misclassification rate (MSE) from the LASSO regression cross-validation procedure was plotted against lambda

**Fig. 4:**
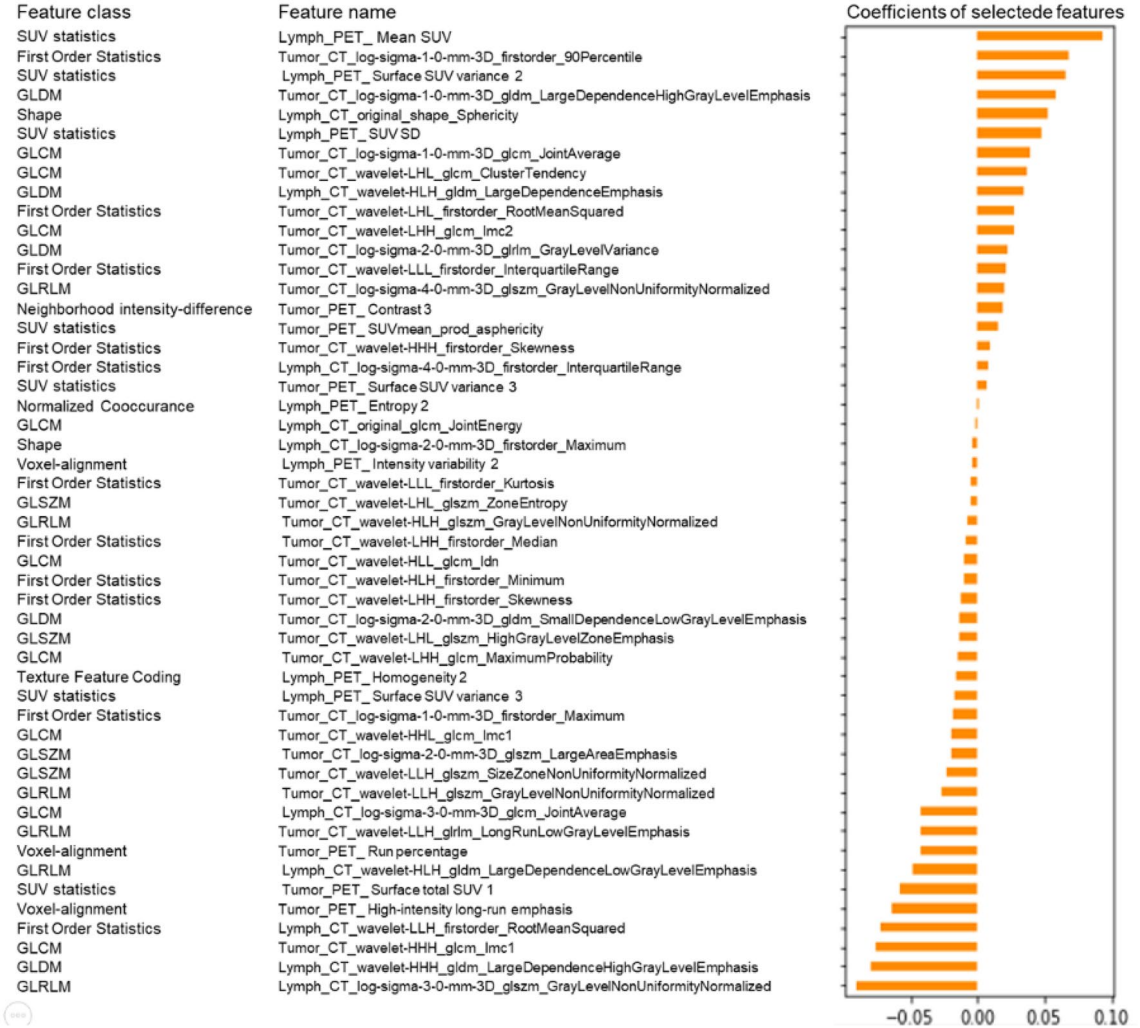
The final 50 selected radiomic features with coefficient rank

The radiomic features were pre-processed, modeled, evaluated, and validated based on the scikit-learn packages (scikit-learn.org) on the Python platform (Swami and Jain 2013). The purpose of feature selection was to recognize a small set of features that are genuinely associated with response from a big pool with ultra-high dimensions. The 2662 radiomic features were ranked based on their importance by applying the Spearman’s relevance to reduce the dimensions and select the optimized features for radiomic modeling. The top 260 most significant radiomic features were selected for reselection analysis. The Spearman’s relevance of the association among the top 260 radiomic features was established using heat maps (Fig. 2). Least absolute shrinkage and selection operator (LASSO) regression analysis was applied to reselect radiomic features with high levels of multicollinearity, thereby eliminating the possibility of overfitting. LASSO with cross-validation (LassoCV) is usually preferable for high-dimensional datasets. The optimal alpha was selected by LASSO with tenfold cross-validation. As the lambda changed from 10^−5^ to 10^−1^, the number of variables entered into the model was reduced, and the absolute values of the coefficients of the variables declined towards zero (Fig. 3a). The LASSO regression model demonstrated the best predictive performance with maximum AUC, while the alpha was 0.00107 with a lambda of 10^–3^ (Fig. 3b). Consequently, 50 highly ranked radiomic features were selected (Fig. 4).

### Radiomics modeling and evaluation

In this study, the features used in the ML algorithms included the 50 radiomic features selected above and seven clinicopathological characteristics. The original datasets were divided into the training and validation set randomly (LN = 283), testing set (LN = 122), and N2-validation set (LN = 57). Based on the three TLPC-, TPC- and LPC-datasets, five independent ML algorithms were trained as classifiers to model the training set, including multi-layer perceptron (MLP), light gradient boosting machine (LightGBM), support vector machine (SVM), gradient boosting decision tree (GBDT), and extreme gradient boosting (XGBoost). To select the best ML algorithms, we typically utilized a six-fold cross-validation on the training set via GridSearchCV method in the hyper-parameter space, with 70% randomly selected to train the models and the remaining 30% used to validate the trained algorithms. XGBoost included 51 estimators with max depth was 6 and the average learning rate was 0.1. Each cross-validation of the ML algorithms might have slightly different optimal parameters. To predict LNM, three prediction models (TLPC, TPC and LPC) were established separately based on the selected clinical factors, tumor PET/CT radiomic features, lymph PET/CT radiomic features, and the combination of the above features using the best ML model implemented in the scikit-learn (version 0.22.1) package. The clinical features selected were age, tumor length, gender, subtype, location, diabetes and smoking. The clinical and radiomics features were input into the prediction models trained to predict LNM in the training cohort. The AUC and the average precision (AP) of the precision-recall (PR) curve were used to identify effective performance quantitatively. The P-value of ROC was assessed using the Delong test.

## Results

### Patient characteristics

The clinical characteristics of the 192 patients with 192 primary tumors and 462 lymph nodes enrolled in the training (LN = 283), testing (LN = 122), and N2-validation (LN = 57) sets are summarized in Table 1. There was total 176 lymph nodes histologically positive (LNM+) and 286 lymph nodes histologically negative (LNM-). There’s statistical difference in the training set between LNM+ and LNM− group. There’s no statistical difference in subtype (*P* = 0.819) and location (*p* = 0.523) in the testing set between LNM+ and LNM− group. There’s no statistical difference in tumor length (*P* = 0.668), diabetes (*P* = 0.862) and smoking (*P* = 0.544) in the N2-validation set between LNM+ and LNM− group.

**Table 1:** Characteristics of the patients in different sets

Characteristics	Total (LN = 462)	Training set (LN = 283)			Testing set (LN = 122)			N2-validation set (LN = 57)		
	LNM+ (176) LNM-(286)	LNM+ (100)	LNM− (183)	*P* value	LNM+ (51)	LNM− (71)	*P* value	LNM+ (25)	LNM− (32)	*P* value
Age (mean ± SD)	63.77 ± 9.13	61.26 ± 8.88	65.49 ± 9.02	< 0.001	60.92 ± 8.90	65.06 ± 7.87	0.008	61.12 ± 7.66	65.59 ± 11.67	0.103
Tumor length	4.57 ± 1.46	4.62 ± 1.51	4.49 ± 1.43	0.477	4.84 ± 1.36	4.54 ± 1.53	0.272	4.41 ± 1.33	4.58 ± 1.63	0.668
Gender										
Female	130 (28.1%)	40(40.0%)	38(20.8%)	0.001	11 (21.6%)	23 (32.4%)	0.182	11 (44.0%)	7 (21.9%)	0.085
Male	332 (71.9%)	60(60.0%)	145 (79.2%)		40 (78.4%)	48 (67.6%)		14 (56.0%)	25 (78.1%)	
Subtype										
ADC	266 (57.6%)	73(73.0%)	90 (49.2%)	0.007	30 (58.8%)	41 (57.7%)	0.819	21 (84.0%)	11 (34.4%)	< 0.001
SCC	182 (39.4%)	24(24.0%)	91 (49.7%)		18 (35.3%)	25 (35.2%)		4 (16.0%)	20 (62.5%)	
LCC	14 (3.0%)	3(3.0%)	2 (1.1%)		3 (5.9%)	5 (7.1%)		0 (0.0%)	1 (3.1%)	
Location										
Right	249 (53.9%)	67 (67.0%)	97 (53.0%)	0.021	26 (51.0%)	32 (45.1%)	0.523	15 (60.0%)	12 (37.5%)	0.095
Left	213 (46.1%)	33 (33.0%)	86 (47.0%)		25 (49.0%)	39 (54.9%)		10 (40.0%)	20 (62.5%)	
Diabetes										
No	429 (92.9%)	97 (97.0%)	163 (89.1%)	0.006	49 (96.0%)	65 (91.5%)	0.296	24 (96.0%)	31 (96.9%)	0.862
Yes	33 (7.1%)	3 (3.0%)	20 (10.9%)		2 (4.0%)	6 (8.5%)		1 (4.0%)	1 (3.1%)	
Smoking										
No	194 (42.0%)	49 (49.0%)	67 (36.6%)	0.046	16 (31.4%)	35 (49.3%)	0.046	13 (52.0%)	14 (43.7%)	0.544
Yes	268 (58.0%)	51 (51.0%)	116 (63.4%)		35 (68.6%)	36 (50.7%)		12 (48.0%)	18 (56.3%)	

*P* value is obtained by using variance analysis between-group

*ADC* adenocarcinoma, *SCC* squamous cell carcinoma, *LCC* large cell carcinoma

### Performance of the ML models

**Fig. 5:**
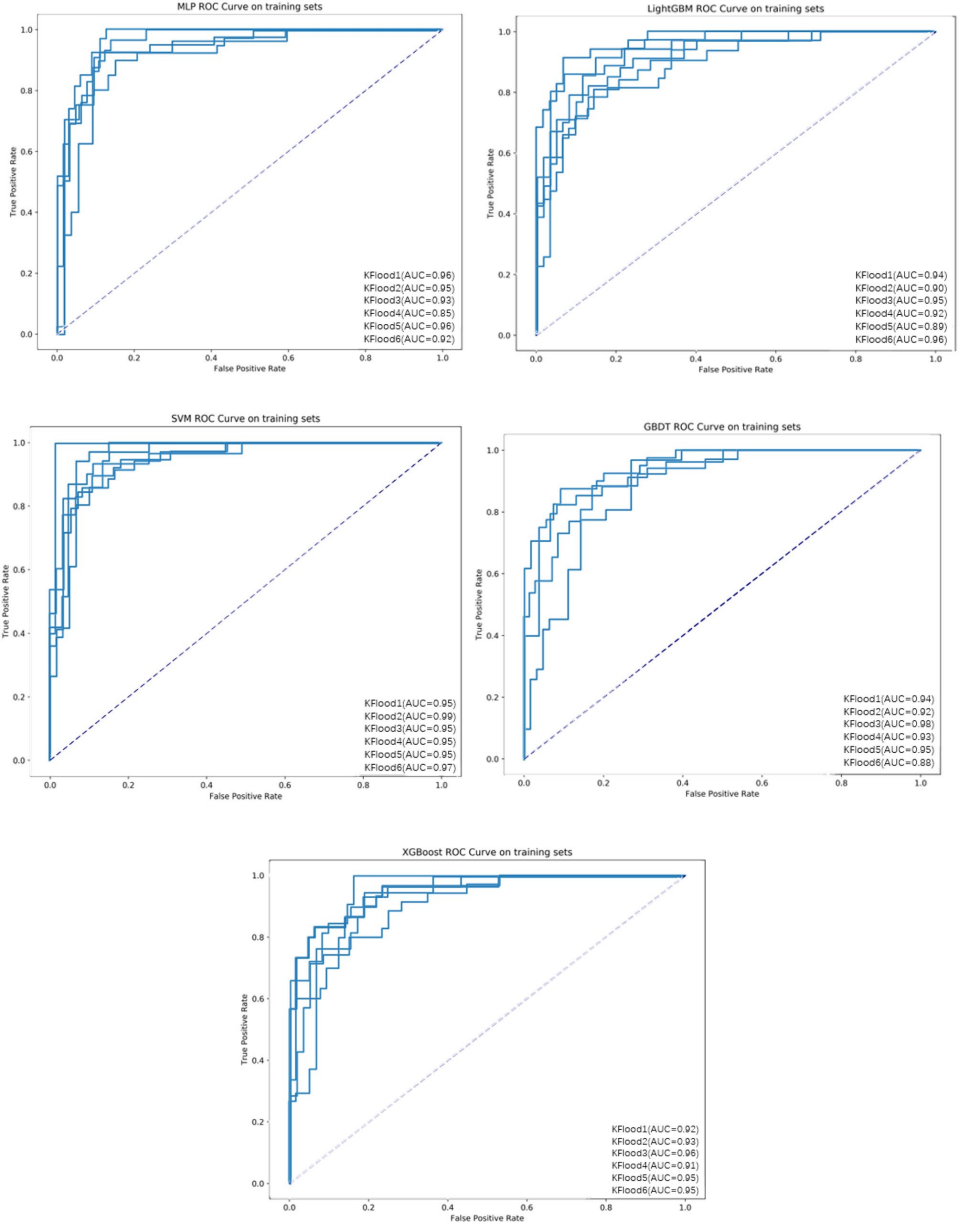
ROC curves of the five ML algorithms for the training set

**Fig. 6:**
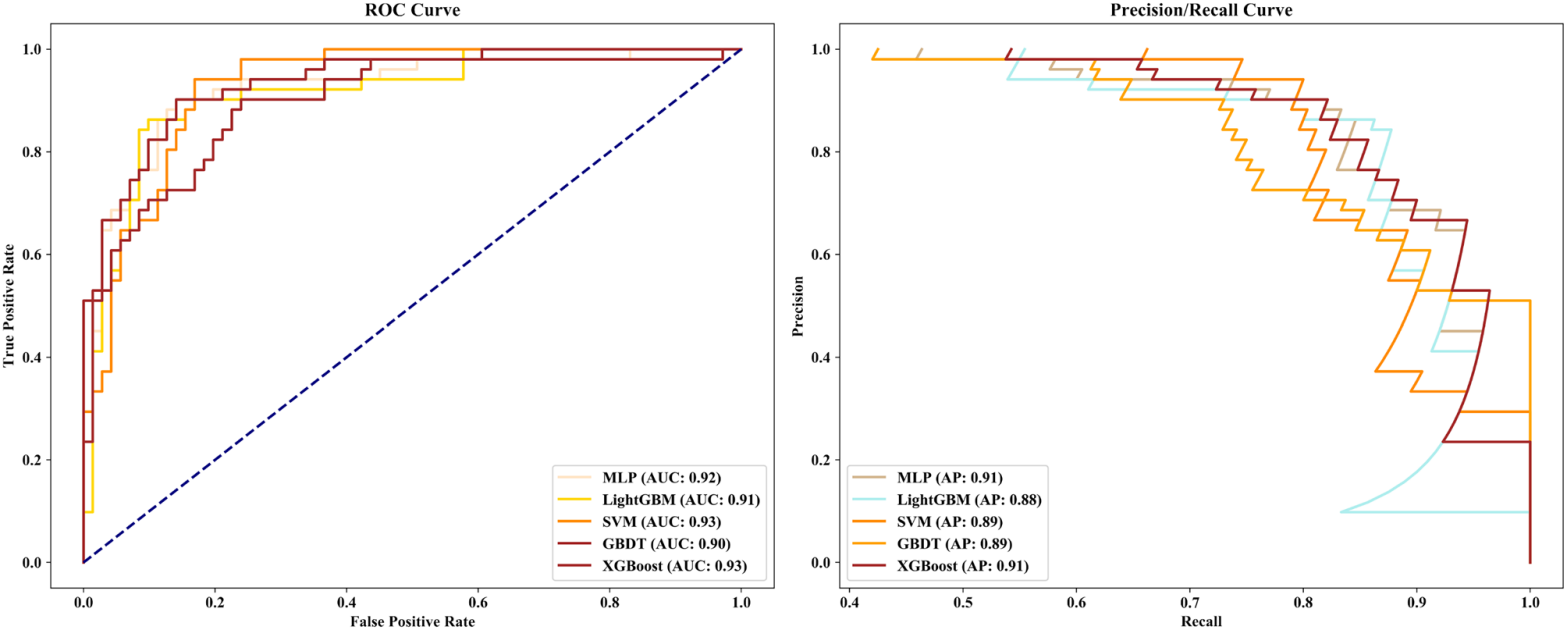
ROC and PR curves of five ML models on the testing set

The performance of the five ML algorithms for the training and validation set after training is shown in Fig. 5. The mean AUCs of the MLP, LightGBM, SVM, GBDT, and XGBoost algorithms were 0.928, 0.926, 0.960, 0.933, and 0.937, respectively, for the validation set. When we evaluated the ML algorithms’ performance on testing set, the SVM and XGBoost algorithms had the same AUC of 0.93, but SVM had poor sensitivity (0.55), whereas XGBoost had an accuracy of 0.85 (specificity: 0.93, sensitivity: 0.75) and AP reached 0.91 (Fig. 6, Table 2). Therefore, we chose the XGBoost algorithm as the classifier for the prediction models due to its optimal performance.

**Table 2:** The five ML models’ detailed diagnostic metrics on testing set

ML models	LNM status	Precision	Recall	F1-score	Support	Accuracy
MLP	(−)	0.82	0.92	0.87	71	0.84
	(+)	0.86	0.73	0.79	51	
LightGBM	(−)	0.81	0.92	0.86	71	0.83
	(+)	0.86	0.71	0.77	51	
SVM	(−)	0.75	0.96	0.84	71	0.79
	(+)	0.90	0.55	0.68	51	
GBDT	(−)	0.80	0.92	0.86	71	0.82
	(+)	0.85	0.69	0.76	51	
XGBoost	(−)	0.84	0.93	0.88	71	0.85
	(+)	0.88	0.75	0.81	51	

### Performance of the prediction models

**Fig. 7:**
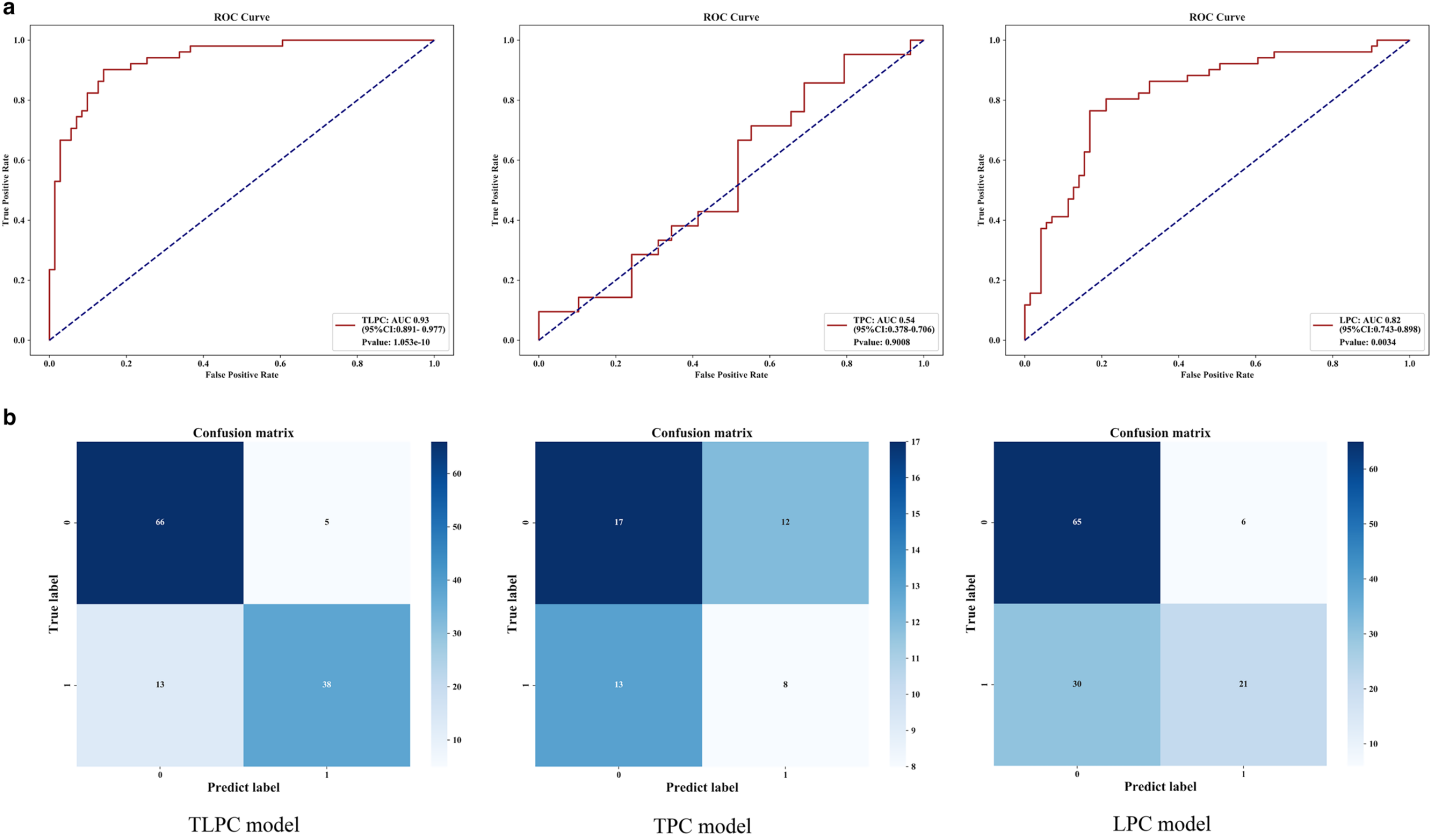
Performance of the three prediction models after application of the XGBoost algorithm to the testing set. **a** ROC curve analyses; **b** Confusion matrices for binary classification

**Fig. 8:**
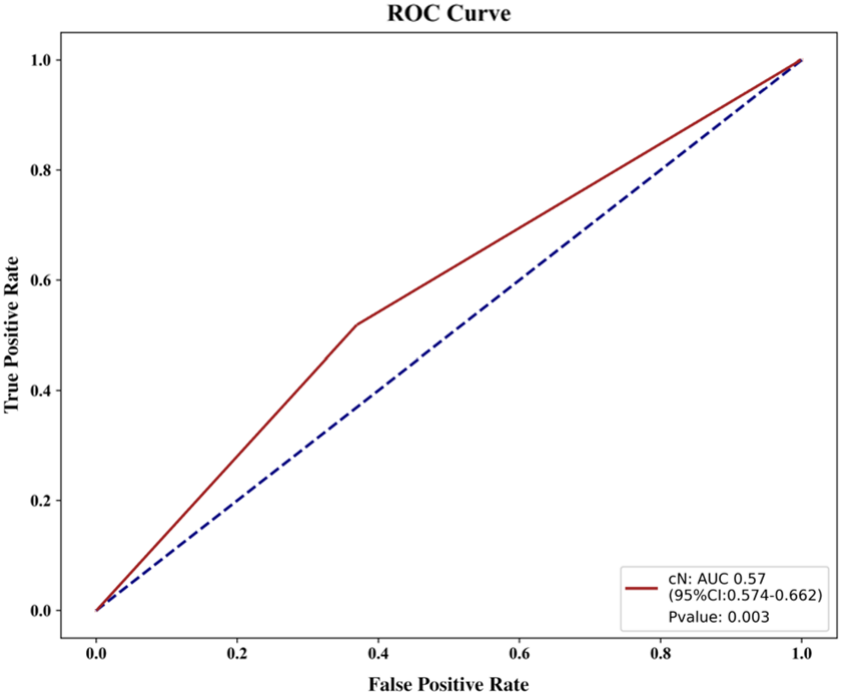
Performance of the cN staging

To compare the effectiveness of TPC, LPC, and TLPC models, we evaluated their results for the testing set (Fig. 7, Table 3). And the performances of the cN staging were also evaluated (Fig. 8). The TLPC model had better values (AUC = 0.93 (95% CI 0.891–0.977; specificity = 0.75; sensitivity = 0.93) than the TPC model (AUC = 0.54; 95% CI 0.378–0.706; specificity = 0.38; sensitivity = 0.59), the LPC model (AUC = 0.82; 95% CI 0.743–0.898; specificity = 0.41; sensitivity = 0.92) and the cN staging (AUC = 0.57; 95% CI 0.574–0.662; specificity = 0.62; sensitivity = 0.52).

**Table 3:** Detailed diagnostic metrics of the 3 prediction models established in this study

Model	LNM status	precision	recall	f1-score	support	Accuracy
On testing set						
TLPC model	(−)	0.84	0.93	0.88	71	0.85
	( +)	0.88	0.75	0.81	51	
TPC model	(−)	0.57	0.59	0.58	29	0.50
	(+)	0.40	0.38	0.39	21	
LPC model	(−)	0.68	0.92	0.78	71	0.70
	(+)	0.78	0.41	0.54	51	
On N2-validation set						
TLPC model	(−)	0.91	0.97	0.94	32	0.93
	(+)	0.96	0.88	0.92	25	
TPC model	(−)	0.53	0.75	0.62	12	0.61
	(+)	0.73	0.50	0.59	16	
LPC model	(−)	0.74	0.78	0.76	32	0.72
	(+)	0.70	0.64	0.67	25

**Fig. 9:**
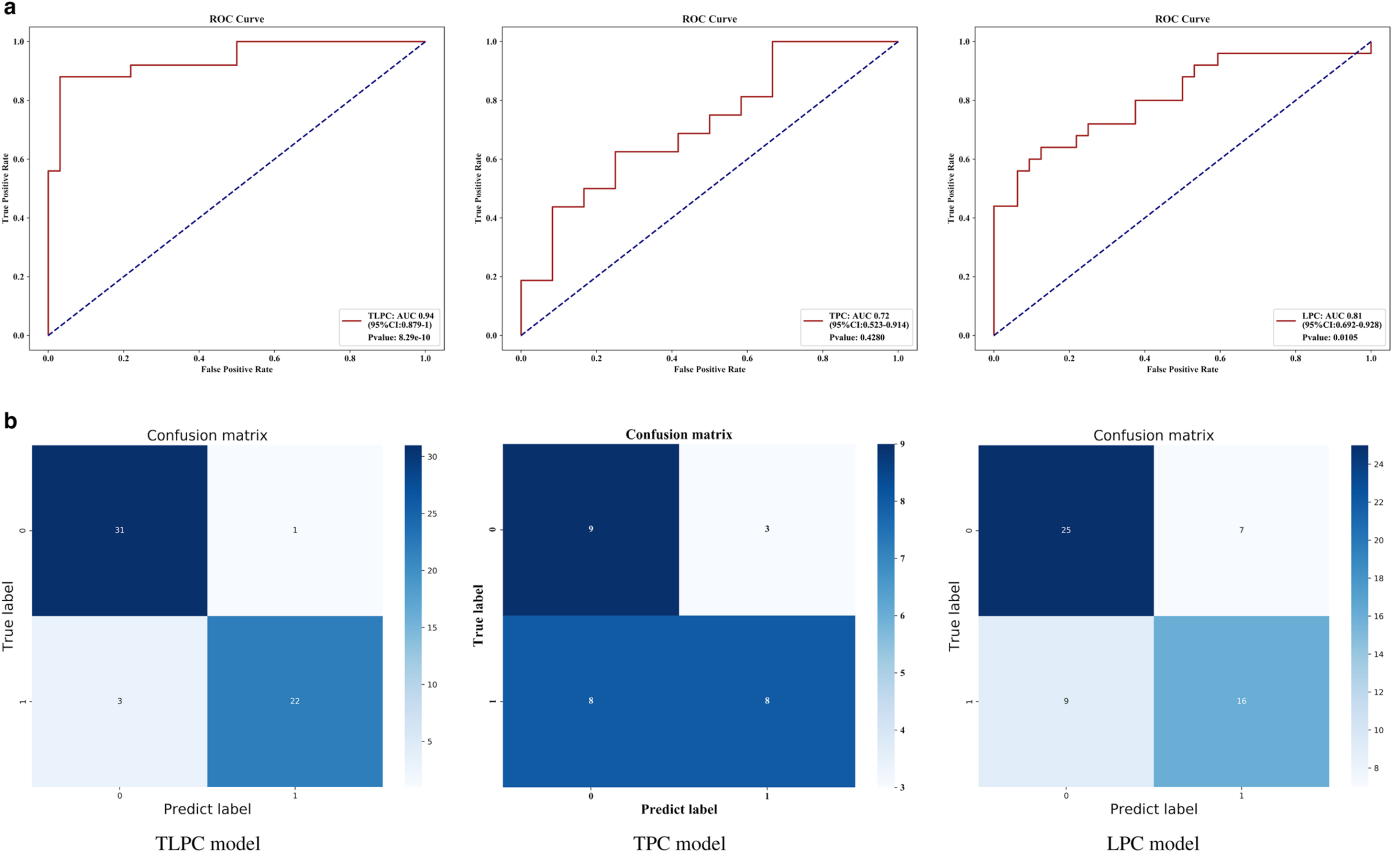
Performance of the three prediction models on the N validation set. **a** ROC analyses; **b** Confusion matrices for binary classification

To evaluate the predicting performance in N2 status of the three models, we applied them to the N2 validation set. The TLPC model yielded the best results, with AUC of 0.94 (95% CI 0.879–1, sensitivity of 0.97, and specificity of 0.88. Detailed diagnostic performance metrics of the other models are summarized in Fig. 9 and Table 3.

## Discussion

Clinical staging of NSCLC before treatment is crucial for developing lung cancer treatment strategies. For resectable NSCLC, a precise N staging before lung cancer treatment helps clinicians decide whether to give neoadjuvant radio/chemotherapy before surgical treatment. Patient age, tumor size, degree of differentiation, and tumor location have been reported as independent risk factors of positive LNM in patients with T1 NSCLC (Pan et al. 2006). Additionally, radiomic features of tumors based on imaging techniques, including PET/CT, which have been shown to have the potential to predict positive LNM in patients with T1 NSCLC (Lv et al. 2021; Chen et al. 2019b; Xiong et al. 2016). Yang el. reported that the CT-based radiomics signature could stratify the risk of N2 metastasis in clinical stage I lung adenocarcinoma (Yang et al. 2019). Carvalho el. combined imaging information based on FDG-PET-radiomics features from tumors and lymph nodes and reported that it helped achieve a higher prognostic discriminative power for NSCLC (Carvalho et al. 2018). Cong et al. built a radiomics model using the venous phase of contrast-enhanced CT and reported that it has potential for predicting LNM in pre-surgical CT-based stage IA NSCLC patients (Cong et al. 2020a). Although the detection of LNM for resectable T2-4 NSCLC is essential for developing surgical or non-surgical treatment strategies, few studies have focused on developing non-invasive methods in predicting LNM in T2-4 NSCLC. Therefore, we developed a model based on PET/CT in predicting LNM in resectable T2-4 NSCLC. Our model predicted the AUC of whole node metastasis to be 93% and that of mediastinal node metastasis to be 82%. For all we know, it’s the first report about predicting LNM of T2-4 NSCLC based on PET/CT.

Accurate N staging of resectable T2-4 NSCLC before lung cancer treatment is important for developing different treatment strategies. Surgical treatment is recommended for NSCLC with N0-1, neoadjuvant chemotherapy should be given pre-operatively for NSCLC with N2 LNM, while surgery is not recommended for those with N3 node metastasis. Our model could improve the efficiency of N-stage prediction and treatment guidance to improve the prognosis of patients. Precise localization of positive lymph nodes can effectively improve biopsy efficiency, avoid unnecessary biopsy, and guide rational clinical diagnosis and treatment (Wiegmann et al. 2018; Liptay et al. 2000). Application of our model can help noninvasive predict the status of the lymph nodes in the mediastinum and pulmonary hilum before treatment to improve the accuracy of pathologic diagnosis, thereby improving the efficiency of N-stage diagnosis and helping in the development of lung cancer treatment strategies.

Although certain meta-analyses of randomized trials of neoadjuvant chemotherapy showed a significant survival advantage over surgery alone, with a hazard ratio of 0.8 that equated to a survival advantage of 5% at 5 years (Burdett et al. 2005; NSCLC Meta-analysis Collaborative Group 2014). Currently, neoadjuvant chemo/radio-therapy is still not the standard process for lung cancer treatment before tumor resection, and only being recommend to the local-invasive or local-metastasis NSCLC, including those with N2-3 node metastasis (Ettinger et al. 2021; Steger et al. 2009). Therefore, our data may also be useful for clinicians to determine whether the neoadjuvant chemotherapy is necessary before tumor resection in lung cancer treatment invasively. For patients with highly suspicious N2 and with > 50% probability of being positive LNM as predicted by the TLPC model but who failed pathological examination, adjuvant chemo/radiotherapy may be given first before surgical treatment to improve their prognosis. Because our model showed great potential for predicting both N1+ and N2+ of T2-4 NSCLC, our data may also have the potential to predict N3+ of T2-4 NSCLC, which would be useful for developing lung cancer treatment strategies or improving the efficacy of lymph node biopsy.

Radiomics provides a reliable tool for noninvasive prediction of N staging (Coroller et al. 2017; Zhu et al. 2019). In recent years, models of pre-operative mediastinal staging incorporating medical images have been widely published, offering more data about different mediastinal staging methods. The summary sensitivity and specificity estimate for the SUV_max_ > 2.5 PET/CT positivity criterion were 81.3% and 79.4%, respectively (Schmidt-Hansen et al. 2014). However, non-invasive prediction of N staging is still difficult in clinical diagnosis. Current prediction performance for node metastasis differs among models due to the various datasets or methods used. A retrospective study based on the primary tumor reported the predictive performance for LNM of the radiomics-clinical model, with AUC values for training and testing of 0.911 and 0.860, respectively (Cong et al. 2020a). However, the prediction model for LNM in NSCLC presented therein mainly focused on T1 NSCLC and was based on radiomics features of primary tumors or lymph nodes separately. In our study, we first assessed the potential of PET/CT to predict positive LNM in patients with T2-4 NSCLC. We also creatively built our model by combining radiomic features of primary tumor and lymph node together. Promisingly, our model achieved the best prediction efficiency of N staging in NSCLC reported to date (AUC peaked at 0.93 with sensitivity of 0.93 and specificity of 0.75). This result suggested that joint assessment of radiomic features of primary tumor and lymph nodes could significantly improve the prediction efficiency for positive LNM in patients with T2-4 NSCLC.

With the rapid development of radiomics, the conversion of digital medical images into mineable high-dimensional data is motivated by the idea that biomedical images contain information that reflects underlying pathophysiology and that these relationships can be revealed via quantitative image analyses (Gillies et al. 2016). Previous studies confirmed that radiomic analyses could improve tumor diagnosis and were capable of predicting clinical phenotypes (Griethuysen et al. 2017). We used five ML classifiers (MLP, SVM, LightGBM, XGBoost, and XGBoost algorithms) and determined that XGBoost performed best in terms of both AUC and AP. XGBoost implements parallel construction of regression trees through multi-threading. Overfitting was prevented by penalizing the model with LASSO (L1) and Ridge (L2) regularization. XGBoost could find the optimal split point efficiently in weighted data sets by using the distributed weighted quantile sketching algorithm, and the algorithm has a built-in cross-validation method at each iteration, thereby eliminating the need to explicitly program the search or explicitly specify the number of enhancement iterations required in a single run. In this study, we applied the XGBoost ML classification model to predict LNM using radiomic features of primary tumor and lymph nodes from pre-treatment PET/CT images in patients with NSCLC. Significantly, the great potential of our predictive model in predicting LNM in lung cancer indicates that the model may also be useful for predicting tumor molecular phenotype, clinical stage, or biological behavior in other cancer types; further, the idea for the model-establishment may also be used to build other evaluation models to assess lung cancer recurrence, distant metastasis, and prognosis.

The heterogeneity of tumors may explain the advantage of evaluating mediastinum LNM by combining the PET-radiomic data of both the primary tumor and the lymph node mechanically. Spatial heterogeneity of tumors in different sites means that the primary tumor may have different molecular phenotypes, imaging features, and metabolism features compared to those of metastatic tumors, and metastatic tumors in different lesions or organs can also have different molecular, imaging, and metabolism features even within the same patient (Chen et al. 2019c; Parker et al. 2020; Pe'er et al. 2021; Klughammer et al. 2018; Schurch et al. 2020; Ozcan et al. 2015; Liu et al. 2018). Therefore, the combination of radiomic features of primary tumor and lymph node may provide more comprehensive information about the lymph nodes, which may be helpful for more accurately diagnosing the N stage of the patient. Herein we demonstrated that the combination of radiomic features of primary tumor and lymph node based on PET/CT improved the LNM diagnostic sensitivity, specificity and accuracy from 0.59, 0.38, and 0.50 of the TPC model and 0.92, 0.41, and 0.70 of the LPC model to 0.93, 0.75, 0.85 of the TLPC model in the testing set. The combination yielded better diagnostic efficacy than most of the existing forecasts, with values much higher than those of previous studies (for which sensitivity, specificity, and accuracy in the testing set ranged from 0.64, 0.94, 0.77 to 0.82, 0.89, 0.85, respectively) (Wang et al. 2017; Cong et al. 2020a, 2020b; Ouyang et al. 2021; Zheng et al. 2021). These findings supported the necessity of comprehensive analysis of each lesion when evaluating the clinical stage of lung cancer patients; this approach may also be necessary for evaluating therapeutic efficacy or prognosis of lung cancer patients.

This study had some limitations. Due to case-collection limitations, this study did not validate the TLPC model in an external cohort. We will continue to collect more datasets from cooperative hospitals. For the ROI segmentation, we used the manual segmentation of the semi-automatic “adaptive brush” tool to obtain the ROIs of primary tumors and lymph nodes. Although the segmentation method is classic and validated as effective, it is time consuming and has inevitable discrepancies due to the manual operation. Some lymph nodes might have a short diameter < 5 mm, while the number of voxels was insufficient for meaningful heterogeneity measurements. Therefore, we selected lymph nodes with a maximum diameter of > 5 mm.

## Conclusion

Our PET/CT-clinical model combined tumor and lymph nodes has the potential to predict LNM in patients with resectable T2-4 NSCLC and therefore helps clinicians in developing more rational therapeutic strategies.

## Data Availability

The data that support the findings of this study are available from the corresponding author upon reasonable request.
